# Genetic architecture and temporal analysis of *Caenorhabditis briggsae* hybrid developmental delay

**DOI:** 10.1371/journal.pone.0272843

**Published:** 2022-08-11

**Authors:** Leonardo Velazco-Cruz, Joseph A. Ross

**Affiliations:** Department of Biology, California State University, Fresno, California, United States of America; Centre National de la Recherche Scientifique & University of Nice Sophia-Antipolis, FRANCE

## Abstract

Identifying the alleles that reduce hybrid fitness is a major goal in the study of speciation genetics. It is rare to identify systems in which hybrid incompatibilities with minor phenotypic effects are segregating in genetically diverse populations of the same biological species. Such traits do not themselves cause reproductive isolation but might initiate the process. In the nematode *Caenorhabditis briggsae*, a small percent of F2 generation hybrids between two natural populations suffer from developmental delay, in which adulthood is reached after approximately 33% more time than their wild-type siblings. Prior efforts to identify the genetic basis for this hybrid incompatibility assessed linkage using one or two genetic markers on chromosome III and suggested that delay is caused by a toxin-antidote element. Here, we have genotyped F2 hybrids using multiple chromosome III markers to refine the developmental delay locus. Also, to better define the developmental delay phenotype, we measured the development rate of 66 F2 hybrids and found that delay is not restricted to a particular larval developmental stage. Deviation of the developmental delay frequency from hypothetical expectations for a toxin-antidote element adds support to the assertion that the epistatic interaction is not fully penetrant. Our mapping and refinement of the delay phenotype motivates future efforts to study the genetic architecture of hybrid dysfunction between genetically distinct populations of one species by identifying the underlying loci.

## Introduction

Species formation involves heritable genetic incompatibilities that will cause biological speciation in the form of hybrid sterility or inviability [1, 2]. A description of how genetic incompatibilities might cause hybrid dysfunction, the Dobzhansky-Muller Incompatibility (DMI) model, posits that new alleles arise at two or more loci in two populations, and that those variants are neutral or beneficial in the background of the populations they arose in [3–5]. Inter-population mating combines those alleles in some hybrid individuals. If those alleles are incompatible, then the hybrid individuals containing both alleles would exhibit a deleterious phenotype. If that phenotype causes reproductive isolation (i.e. sterility or inviability), then the two populations would be classified as two biological species. While gene flow is reduced between those species, additional genetic variants could accumulate, some of which might reinforce a species barrier. This process has been termed the “snowball effect” [6]. This process can complicate efforts to identify the original incompatibility after enough time has passed. With the goal of identifying the genetic variants that initiate the speciation process, the study of incipient speciation is critical in part because it avoids potential complications involving the snowball effect [6].

An obstacle to understanding the genetic basis of speciation has been a paucity of cases in which incipient speciation has been elucidated at the molecular level [7, 8]. Notable cases include inter-population hybrids of the nematodes *Caenorhabditis elegans* and also in *C*. *tropicalis*, where toxin-antidote (TA) elements reduce hybrid fitness [9–12]. Toxin-antidote elements are selfish gene pairs in which a toxin is present in all offspring, and only those offspring that inherit a linked antidote gene are rescued from the toxin element’s lethal effects [13–15].

*C*. *briggsae*, a relative of *C*. *elegans*, occupies an important niche in the study of the genetics of speciation and of development. Natural populations (“wild isolates”) of *C*. *briggsae* collected from around the world form at least three genetically distinct phylogeographic clades (temperate, tropical circles of latitude, and equatorial) that correlate with isolate latitude [16–18]. The AF16 and HK104 wild isolates are routinely used in *C*. *briggsae* genetic studies, the genome assembly of this species was originally based on the AF16 sequence [19], and more recent work using the isolates QX1410 and VX34 have improved the quality of the genome assembly [20]. *C*. *briggsae*, like *C*. *elegans*, is a primarily selfing species, having an androdioecious mating system (separate male and hermaphrodite individuals, where males are rare in nature). Thus, wild isolates tend to be homozygous throughout the nuclear genome [19]. AF16 and HK104 are ~0.6% divergent in the nuclear genome, having a single nucleotide polymorphism (SNP) on average every 163 base pairs [21, 22], making AF16-HK104 hybrids useful for genetic mapping. *C*. *briggsae* research also benefits from a suite of genetic, genomic and molecular tools [19, 22–25].

An increased emphasis in studying speciation genetics has recently occurred in the *Caenorhabditis* genus because viable hybrids can be produced between certain species pairs, including *C*. *briggsae*–*C*. *nigoni* [26–30], *C*. *remanei*–*C*. *latens* [31, 32], and *C*. *nouraguensis–C*. *becei* [33], and some of these crosses produce fertile hybrids. Some *Caenorhabditis* species also contain hybrid incompatibilities segregating within the same species, like in *C*. *nouraguensis*, which exhibits a hybrid lethal cytoplasmic-nuclear incompatibility [34].

*C*. *briggsae* populations have been reported to suffer from more mild hybrid incompatibilities. The AF16 and HK104 wild isolates have experienced mito-nuclear coevolution that causes dysfunction when the mitochondrial and nuclear genomes are separated in inter-isolate hybrids [35]. AF16-HK104 hybrids also exhibit outbreeding depression in the forms of increased embryonic lethality and developmental delay [19, 36, 37], the latter of which is the focus of the present study. Developmental delay in AF16-HK104 F2 hybrids has been described as a difference in developmental stage at 48 hours post laying, where the parental isolates and their wild-type F2 hybrids reach the L4 developmental stage in approximately two days, while delayed F2 siblings require an additional day to reach the same stage [19, 37]. Thus, *C*. *briggsae* offers a rich opportunity to study the genetic causes of intra-specific genetic incompatibilities and possibly the onset of species formation.

Initial experimental evidence suggested that developmental delay results from a hybrid genetic incompatibility between AF16 and HK104 alleles. Selfing or crossing individuals from the same isolate rarely produced developmentally delayed offspring, with F2 delay frequencies ranging between 0.00–0.02 [12, 37]. However, when the two wild isolates are mated, either crossing or selfing the AF16-HK104 hybrid F1 individuals produced F2 delay frequencies between 0.14 and 0.21, and the dependence of delay on cross direction suggested that this phenotype depends on a maternal effect [37]. A subsequent study reported an F2 delay frequency of 0.21 and showed that F1 backcrosses to AF16 males produced approximately double the delay frequency (0.39 in the BC1 generation), which suggested that delay is caused by an HK104 maternal-effect toxin-antidote element [12] and not a multi-locus AF16-HK104 epistatic interaction as initially proposed. However, none of these observed frequencies strictly adhere to expectations for TA elements (0.25 frequency for F1 selfing; 0.5 frequency for BC1), which raises the possibility that modifier loci or other factors result in incomplete penetrance of the delay trait.

Our long-term goal is to identify the genetic basis for this intra-species hybrid incompatibility. The initial bulk segregant mapping study showed that delayed AF16-HK104 F2 hybrids tend to be overrepresented for AF16 alleles at two loci on chromosome III, at 10.1 and 12.2 Mbp [19]. The same study suggested that marker transmission ratio distortion favoring AF16 alleles in the central recombination domain of III (spanning from 4.7 to 10.8 Mbp on the chromosome assembly) might have been caused by unintended artificial selection against the delay phenotype caused by a TA element in HK104 [19]. A subsequent study identified linkage of delay to a marker at 4.01 Mbp on III [12]. Thus, the delay locus has only been broadly mapped. Here, we have assessed the temporal onset of F2 hybrid developmental delay to produce a more accurate definition of this phenotype, and we then combined this phenotype description with genotypes of delayed AF16-HK104 F2 hybrid individuals at markers spanning chromosome III to refine the delay locus.

## Materials and methods

### Nematode strains and husbandry

The *C*. *briggsae* wild isolate strains AF16 and HK104 were obtained from the Caenorhabditis Genetics Center. ZZY00020, which is AF16 containing a GFP transgene [myo-2::GFP], was obtained from Z. Zhao [29]. Populations were maintained in 20° incubators on nematode growth medium (NGM) agar plates seeded with cultures of the *Escherichia coli* strain OP50 according to standard practice [38]. A digital peristaltic pump was used to dispense agar medium, and a digital dispensing pipettor was used to dispense volumes of OP50 culture onto plates. These measures were taken to ensure that environmental conditions (agar thickness; food availability) were as consistent as possible from plate to plate.

### Hybrid backcrosses

P0 generation AF16 hermaphrodites were self-sperm depleted by moving them onto new NGM plates daily until no self-offspring were produced in a 24 hour period, rendering a hermaphrodite functionally self-sterile (“pseudofemale.”) Each pseudofemale was mated to 3–5 HK104 males. After 24 h, if mating occurred, then the P0 pseudofemale was producing F1 offspring. All of the P0 males and the P0 pseudofemale were then removed from the plate. At approximately 48 h, L4 stage F1 hermaphrodites (virgins) were isolated on separate plates, self-sperm depleted, and then backcrossed to new HK104 males to produce BC1 offspring.

### Developmental profiling and micrography

Synchronized F2 individuals were collected by simultaneous embryo production from multiple adult AF16-HK104 F1 hybrid hermaphrodites, multiple adult AF16 hermaphrodites (control), and multiple adult HK104 hermaphrodites (control). Each of the three populations was housed on its own NGM agar plate for two hours at the same time, during which the adults laid fertilized embryos onto the agar substrate. At the end of two hours, all of the adults were removed. Embryos present on each of the NGM plates were thus the same developmental age (within two hours). Embryos were singled onto new NGM plates and micrographed at 24, 36, 48, 52, 58, and 72 hours post-laying (hpl). To avoid potential artifacts (e.g. change in nematode length, harming the nematode) that could be introduced by anesthetizing and mounting worms on glass slides repeatedly during their development, we micrographed each nematode on its NGM agar plate at room temperature using a Zeiss AxioCam MRm digital camera attached to a Zeiss Discovery.v8 dissecting stereoscope at 8x magnification.

The micrographs were manually analyzed with ImageJ [39] using the segmented line tool to measure the anterior-posterior length (in arbitrary units) of each individual at each timepoint. Arbitrary units were then converted to metric lengths by using ImageJ to measure distances on a micrograph of a hemocytometer grid. We then used these data to calculate the micrometer per arbitrary unit ratio, and we multiplied this value by the arbitrary unit length from each micrograph. Using nematode total length to assess development regardless of developmental stage, which is the standard metric for reporting development, is intended to circumvent concerns about qualitative assessment of nematode developmental stage [40].

### Developmental delay phenotyping

AF16-HK104 F2 hybrid developmental delay has been described as presence of L2/L3 larvae at 48 hours, while wild-type siblings are L4 larvae at that point [37]. Here, we described a worm as delayed if at 58 hpl it remained an L4 larva, based on visual identification of L4.3–L4.7 stage vulval morphology [41].

### Genotyping

Each individual nematode was isolated in a PCR tube containing 38 μL of single-worm lysis buffer (50 mM KCl, 10 mM TRIS pH 8.3, 2.5 mM MgCl2, 0.45% nonidet P-40, 0.45% Tween-20, 0.01% gelatin) and 2 μL of 20 mg/mL proteinase K. Each worm was then frozen at -80° and digested for 60 m at 60°. The proteinase K was inactivated by incubation at 95° for 15 m, and then the single worm lysate was immediately used as PCR template.

To genotype nuclear alleles, an amplified fragment length polymorphism (AFLP) analysis was used. Nuclear genome insertion-deletion (indel) markers that distinguish AF16 and HK104 alleles were amplified by polymerase chain reaction (PCR) using published primer sequences [22]. Each 10 μL reaction contained 0.25 U OneTaq DNA polymerase (New England Biolabs), 1x reaction buffer, 0.2 mM each dNTP, either 5 ng of purified DNA template (at 5 ng/μL) or 1 μL of single worm lysate or 1 μL of water, and 1 μM each of the forward and reverse primers. All amplifications occurred in a Bio-Rad T100 thermal cycler using the following cycle sequence: 95° 2 min, 30 cycles of 95° 1 min, 45° 1 min, 68° 2 min, followed by a final 68° 5 min extension.

All amplicons were separated by electrophoresis in type III loading buffer modified by omitting xylene cyanol [42] on 2% agarose gels containing ethidium bromide. Electrophoresis was perfomed in 1x TRIS-acetate EDTA buffer. Promega 100 bp ladder (G2101) was loaded as a molecular weight standard. Digital micrographs of gels were obtained using an AlphaImager HP system (Alpha Innotech).

## Results

### Developmental profiles of F2 hybrids

**Fig 1:**
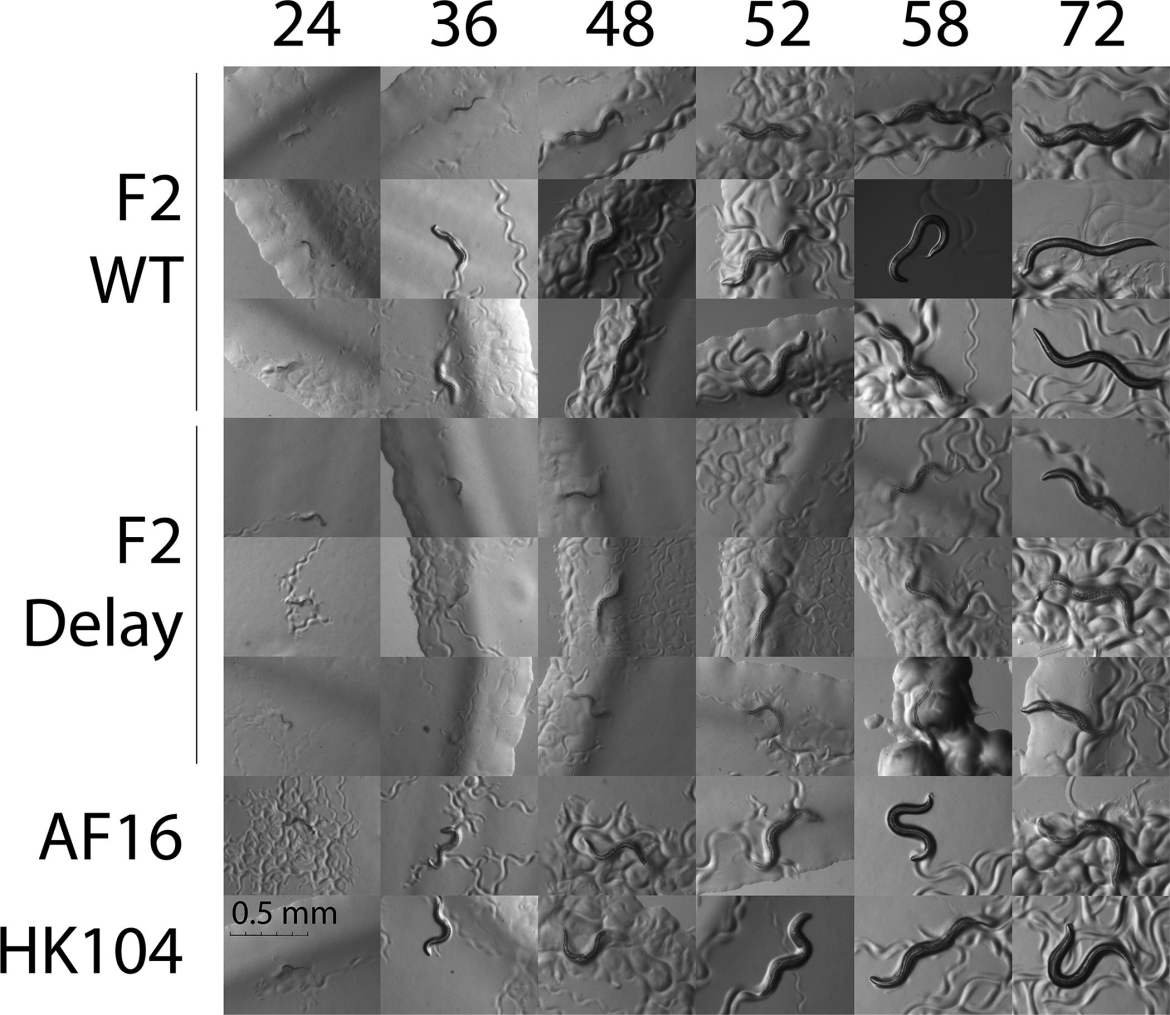
Micrographs of wild-type and delayed individuals. Micrographs of nematodes on NGM agar plates were acquired at the number of hours post-laying (hpl) listed above. Three example wild-type F2 and three developmentally delayed F2 are shown, along with one AF16 and one HK104 individual. Scale bar in the bottom-left panel: 0.5 mm.

The presence of delay in F2 hybrids suggests that there might be a discrete point in time at which typical development either pauses or begins to lag. To characterize the temporal process of developmental delay, 66 F2 hybrid siblings, 22 AF16 and 22 HK104 individuals were micrographed across larval development. Fig 1 shows micrographs of eight example worms across development. S1 Table contains the length of each of the 110 individuals at each timepoint, and all of the original image files are available at 10.6084/m9.figshare.20085863.

In general, wild-type F2s and wild isolates became L4 stage larvae between 36 and 48 hours post-laying (hpl), were still L4 larvae at 52 hpl, and developed into self-fertile adults by 58 hpl. In contrast, delayed F2s did not reach the L4 larval stage until between 48 and 52 hpl, were still L4 larvae at 58 hpl, and became self-fertile by 72 hpl.

**Fig 2:**
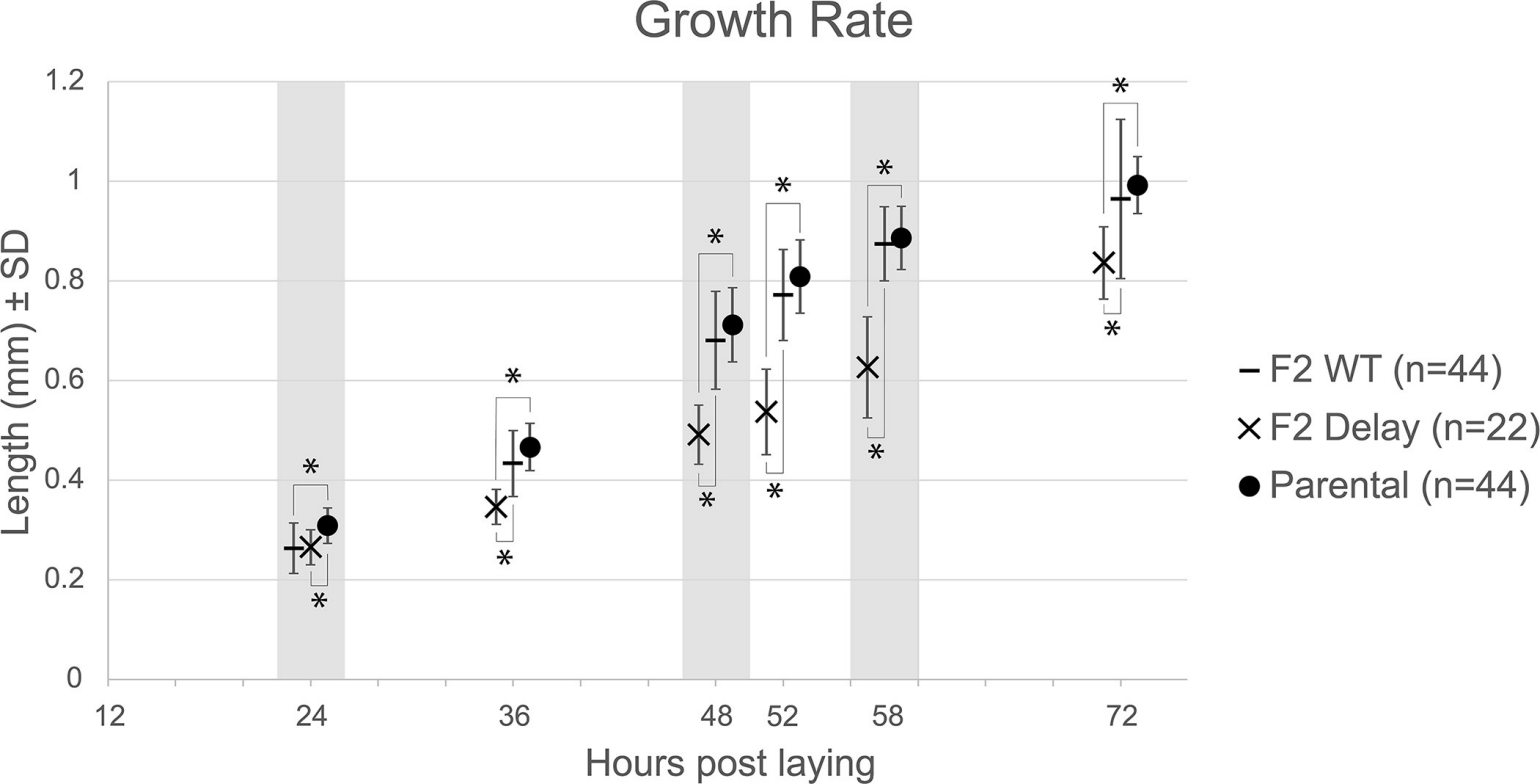
Development of F2 hybrids. Nematode total length is plotted against the number of hours elapsed after each embryo was deposited by a hermaphrodite onto an NGM agar plate. The average length of delayed F2 nematodes is plotted as an X; the average length of wild-type F2 siblings is plotted as a dash (horizontal line). The average length of the combination of the AF16 and HK104 parental isolates is plotted as a circle. Vertical lines depict one standard deviation. Alternating background shading is used to distinguish data collected at 24, 36, 48, 52, 58, and 72 hours post laying, at which point every individual had reached adulthood. The data series at each timepoint have been jittered on the x-axis for clarity. Statistical analyses were conducted to identify significant differences between the parental and F2 wild-type populations, the parental and F2 delayed populations, and the F2 wild-type and delayed populations. Brackets indicate statistically different comparisons (Bonferroni-corrected Student’s T-tests, alpha 0.05; see Table 1 for p-values).

The lengths of the P0 parental, wild-type F2 and delayed F2 populations are plotted in Fig 2. The AF16 and HK104 populations were significantly larger than the delayed F2 population at every timepoint (Table 1), and the wild-type F2 population was significantly larger than their delayed F2 sibling population at all timepoints except 24 hpl.

**Table 1:** Statistical comparisons of F2 hybrid lengths.

Pop-1	Pop-2	24	36	48	52	58	72
P0	F2 (WT)	5.70E-06*	8.43E-03	9.88E-02	3.76E-02	4.29E-01	2.87E-01
P0	F2 (Delay)	2.69E-05*	2.49E-09*	5.24E-14*	2.82E-13*	1.18E-11*	3.24E-05*
F2 (WT)	F2 (Delay)	8.43E-01	2.49E-09*	5.24E-14*	2.82E-13*	1.18E-11*	3.24E-05*

Unpaired two-tailed t-tests identified pairs of populations (“Pop-1” and “Pop-2”) with different lengths at the number of hours post-laying listed in the top row (24–72 hpl). The “P0” population combines all AF16 and HK104 individual measurements.

* indicates timepoints at which each population pair significantly differed in size; the p values are reported. Significance was assessed following Bonferroni correction. For comparisons with significant differences, Pop-1 had a larger average length than Pop-2.

### Producing developmental delay with F1 backcrossing

If homozygosity for AF16 alleles on chromosome III is required to produce developmental delay, then backcrossing an F1 hybrid to HK104 should not produce any delayed offspring. Results from this cross have never been reported; we found that zero of 34 BC1 hybrids exhibited developmental delay, as expected. This finding supports the conclusion that homozygosity for AF16 is necessary to produce developmental delay, as previously suggested, and is consistent with presence of an HK104 toxin-antidote element [12, 19, 37].

### F2 mapping

**Fig 3:**
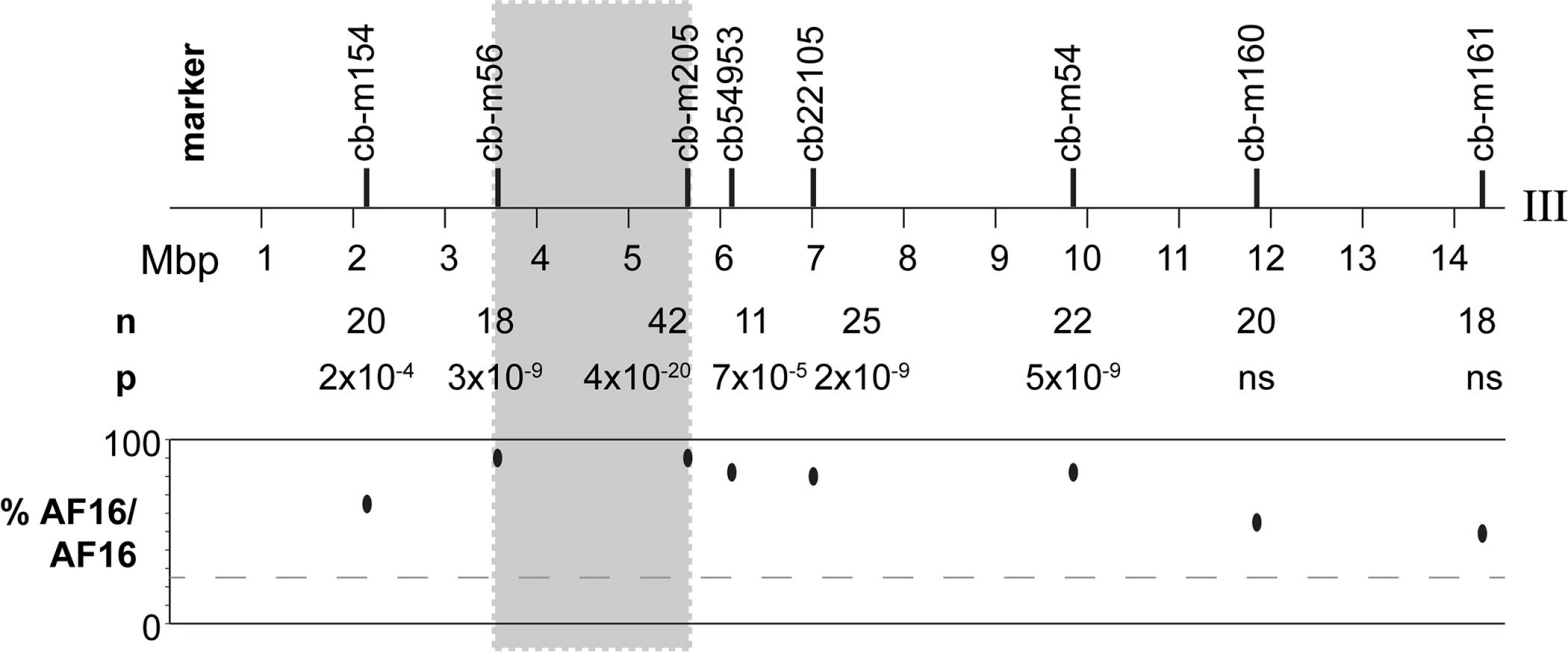
F2 genotypes across chromosome III. The percent of F2 genotypes homozygous AF16 at each marker in developmentally delayed F2 hybrids is plotted on the y-axis (“% AF16/AF16”). The total number of typed individuals (n) at each locus is provided above the plot, along with the p value (chi-square, Bonferroni-corrected) of the difference between expected and observed genotype values. “ns” indicates not significant p values. The gray line at 25% AF16 homozygosity denotes the Mendelian expectation for AF16 homozygosity in F2 hybrids. The gray shaded box indicates the two markers with maximum allele fraction deviation.

To collect F2 individuals for mapping the chromosome III interval associated with delay, several AF16-HK104 F1 hybrid hermaphrodites were selfed to produce 2,622 offspring, of which 515 were developmentally delayed (19.6%). 176 of these delayed F2 individuals were genotyped at one of eight AFLP loci on chromosome III. We used Bonferroni-corrected chi-square analysis to compare these genotypes with the Mendelian expectation in the F2 generation of a 1:2:1 genotype ratio (AF16 homozygous: heterozygous: HK104 homozygous). Delayed individuals exhibited significant deviation toward homozygosity for AF16 alleles at loci in the middle of chromosome III (Fig 3), with a maximum deviation of 88% homozygosity for AF16 at marker cb-m205. In these delayed F2 individuals, six markers revealed significant bias toward homozygous AF16 genotypes. The two markers with non-significant p-values are located near the chromosome III right telomere. These data confirm the presence of a delay locus on III around 3.572–5.649 Mbp.

### Evaluating the accuracy of delay frequency observations

To evaluate the possibility that some F1 offspring of self-sperm depleted hermaphrodites crossed to males might be self-offspring, and thus influence the observed frequency of F2 developmental delay, we self-sperm depleted four HK104 hermaphrodites using the traditional approach (Materials and Methods). We then crossed each with ZZY00020 males, which are AF16 with a GFP transgene. In total, the four hermaphrodites produced 114 offspring after mating. To evaluate the provenance of each offspring, fluorescence microscopy was used to phenotype the presence of GFP. All 114 offspring were fluorescent, indicating that none of the 114 offspring were self-progeny of the HK104 parental hermaphrodite. Thus, even if the process of self-sperm depletion happens not to completely exhaust a hermaphrodite of self-sperm, the number of remaining self-sperm would not be large enough to substantially influence the observed frequency of developmental delay.

## Discussion

### Developmental regulation in *Caenorhabditis*

Developmental delay is a widely reported phenomenon in hybrids [e.g. 43–48]. In this study, we have continued to refine our understanding of the phenotype and the genetic architecture of hybrid developmental delay in *C*. *briggsae* [12, 19, 36, 37]. Although no genetic variants segregating in nature have yet been suggested to cause postembryonic hybrid developmental incompatibilities in the close relative *C*. *elegans*, the process of postembryonic development has been more extensively studied there. In early studies, the lengths of nematodes were measured and correlated with larval molts. This demonstrated that wild-type growth does not involve severe pauses, including during molting [49]. Later studies showed that *Caenorhabditis* larval development is controlled by heterochronic genes [50]. Mutations in these genes can cause accelerated or delayed development [51]. Because developmental delay is a common effect of mutations in *Caenorhabditis*, future efforts to identify the genetic basis of AF16-HK104 hybrid developmental delay should benefit from the temporal description of developmental delay that we provide here. We have found that developmental delay is evident across much of larval development, with a significant decrease in total length between 36 and 72 hours post laying compared to wild-type siblings. Even delayed hybrids routinely become self-fertile adults and are no longer casually distinguishable by eye from their wild-type F2 siblings. We find that delay is easiest to detect by eye at 58 hpl, when wild-type F2 are adults and their delayed F2 siblings are still predominantly L4 larvae. Although we chose to use worm total length as a proxy for development [40], this is not the same approach as identifying developmental stages. Some genetic factors, and thus potentially some incompatibilities, can alter body size irrespective of developmental stage [52, 53]. Thus, it will be valuable in future to compare both size- and developmental stage-based phenotyping.

### Genetic architecture of delay

Because the AF16 and HK104 wild isolates do not exhibit the developmental delay phenotype [37], delay might result from hybrid dysfunction involving a negative epistatic interaction between divergent alleles at two or more loci. Absence of delay in AF16-HK104 F1 hybrids indicates that this phenotype does not result from a dominant epistatic interaction between AF16 and HK104 alleles [37]. The presence of a maternal effect hybrid incompatibility [37] raised the possibility that a toxin-antidote (TA) system causes developmental delay. Such selfish genetic elements have been documented in *Caenorhabditis*, including the *zeel-1*/*peel-1* system in *C*. *elegans* [9, 10] and recently in *C*. *tropicalis* and in *C*. *briggsae*, where this developmental delay phenotype has been reported to be caused by a TA element present in HK104 [12].

Here, we have interrogated the genetic architecture of F2 hybrid developmental delay, which involves homozygosity for AF16 alleles at a locus on chromosome III [12, 19, 37]. The broad chromosome III interval has never been systematically mapped. Initial mapping efforts genotyped two loci on chromosome III, at 10.1 and 12.2 Mbp [19]. In the same study, patterns of marker transmission ratio distortion suggested that the delay locus is in the central recombination domain of III, which spans 6.1 Mbp (from 4.7–10.8 Mbp) on the chromosome assembly and thus comprises almost half of the chromosome [19]. The most recently published mapping effort, which confirmed linkage of the delay locus on III, genotyped a single marker at 4.0 Mbp [12]. Thus, the current understanding is that chromosome III harbors an HK104 maternal effect TA element that causes developmental delay in offspring homozygous AF16 at the TA locus.

Using eight genetic markers spanning III, we find here that the region of strongest association exists between 3.572 and 5.649 Mbp (Fig 3, gray box), with the *caveat* that an association peak could exist beyond the boundaries of this interval, adjacent to the flanking markers. At the markers defining the minimal interval, 88% of genotypes from delayed individuals were AF16/AF16. At both markers, no delayed worm contained an HK104/HK104 genotype. Thus, developmental delay is often, but not always, associated with AF16 homozygosity at these two chromosome III loci. This interval also contains the PCR genotyping assay at 4.0 Mbp that was previously used to map delay, at which 1 of 41 delayed individuals did not contain a homozygous AF16 genotype [12]. Genetic mapping is notoriously difficult in the central recombination domains in *C*. *elegans* and *C*. *briggsae*, where crossing-over rarely occurs [19, 20, 54]. Thus, absence of a perfect genotype-phenotype correlation could be interpreted in at least two not mutually exclusive ways. First, we might not have genotyped a marker perfectly associated with the delay phenotype. Also, it is conceivable that multiple genetic interactions between AF16 and HK104 alleles could result in similar developmental delay phenotypes.

The possibility that hybrid developmental delay exhibits incomplete penetrance should also be considered. Mendelian principles suggest that a TA element present on HK104 chromosome III should produce 25% delay when an AF16-HK104 F1 hermaphrodite self-fertilizes, because all the embryos will contain the HK104 allele-derived toxin, and 25% of the F2 offspring should be homozygous AF16 on III and thus not produce any antidote. For the *C*. *briggsae* developmental delay phenotype, lower delay frequencies of around 20% have been reported by two other independent research groups [12, 37], and the cause for this lower frequency is not known. We report here the largest dataset measuring the developmental delay frequency produced by selfing AF16-HK104 F1 hybrids: our observation of 19.6% delay among 2,622 F2 individuals is statistically significantly different from 25% expected for a completely penetrant TA element (chi-square, p < 3E-10).

Potential causes of incomplete penetrance include the presence of modifier loci and environmental effects [55]. It is conceivable that an unknown modifier locus attenuates the effect of the chromosome III TA genotype. For example, if a dominant HK104 allele necessary for producing developmental delay exists on another autosome, then when 25% of self-offspring from an AF16-HK104 F1 are homozygous AF16 on III, 75% of those F2 (19.75% of all F2) would be homozygous HK104 or heterozygous at the unlinked modifier locus. It would be valuable to conduct genome-wide mapping in F2 populations to identify potential modifier loci. With respect to potential environmental influences, future efforts might explore whether hybrid developmental delay is temperature-sensitive, as has been suggested for another TA element in *Caenorhabditis* that differs in expressivity depending on temperature [12] and also in *Tribolium castaneum*, where both genetic background and temperature influence the time of hybrid death caused by a Medea element [14]. Reduced penetrance of other incompatibility factors, such as *Wolbachia* bacteria, has also been observed in *Drosophila*, possibly as a result of ongoing genetic co-evolution [56–58].

An experimental artifact could also potentially account for some deviation of the observed delay frequency from the expected frequency for a TA element. Any residual self-sperm in the pseudofemales used for crosses would reduce the observed delay frequency [12]. Our experimental results that zero of 114 progeny of a cross between ZZY00020 males and HK104 self-sperm depleted hermaphrodites were fluorescent suggest that even if rare self-sperm are retained by functionally self-sterile hermaphrodites, not enough self-sperm is present to cause the magnitude of effect necessary to bias the experimental measurement of developmental delay frequency. Also, in every case where we used self-sperm depleted P0 hermaphrodites to generate AF16-HK104 F1s, each of those selfed F1 hermaphrodites always produced some delayed F2 offspring. Thus, none of the P0 pseudofemales we used produced unexpected F1 self-offspring, because F1 self-offspring would not have generated F2 hybrid developmental delay.

## Conclusion

Our mapping data agree with the model that homozygosity for an AF16 allele in the middle of chromosome III is necessary to produce hybrid developmental delay and that delay is caused by a maternal-effect HK104 TA element [12]. By monitoring the growth of individual siblings from embryo to adulthood, we have refined the definition of the AF16-HK104 hybrid F2 developmental delay phenotype. At the present temporal resolution, it is not yet possible to conclude whether a particular larval stage is elongated, which might implicate misregulation of a heterochronic gene. However, the developmental lag of F2 delayed individuals, compared to AF16, HK104, and wild-type F2 siblings at every timepoint beyond 24 hours post-laying (Fig 2), supports the interpretation that hybrid developmental delay is not limited to a particular larval stage. These results reinforce the value of using the genetically diverse wild isolates of *C*. *briggsae* to study the genetic basis of a hybrid dysfunction phenotype that might represent one of the early events in the evolution of reproductive isolation and speciation.

## Supporting information

S1 Table Numerical values of nematode lengths. This.csv file contains the individual nematode lengths from micrographs of *Caenorhabditis briggsae* individuals at various hours post laying (hpl: 24, 36, 48, 52, 58 and 72). The first column contains the unique alphanumeric identifier of each individual. The second column provides the population type (“F2” for AF16-HK104 F2 hybrid, “AF16” and “HK104”), the next six columns contain their lengths (in mm) at 24, 36, 48, 52, 58 and 72 hours post laying, and the final column reports each individual’s phenotype: wild-type (“WT”) or developmentally delayed (“Delay”).
(CSV)
